# Early-onset pancreatic cancer: a review of molecular mechanisms, management, and survival

**DOI:** 10.18632/oncotarget.28242

**Published:** 2022-06-15

**Authors:** Mark B. Ulanja, Alastair E. Moody, Bryce D. Beutler, Daniel Antwi-Amoabeng, Ganiyu A. Rahman, Olatunji B. Alese

**Affiliations:** ^1^Christus Ochsner Saint Patrick Hospital, Lake Charles, LA 70601, USA; ^2^Department of Anesthesiology, University of Utah, Salt Lake City, UT 84112, USA; ^3^Department of Radiology, University of Southern California, Keck School of Medicine, Los Angeles, CA 90033, USA; ^4^Department of Surgery, University of Cape Coast, School of Medical Sciences, Cape Coast, Ghana; ^5^Department of Hematology and Oncology, Winship Cancer Institute, Emory University, Atlanta, GA 30322, USA

**Keywords:** pancreatic cancer, gastrointestinal malignancy, epidemiology, pancreatic adenocarcinoma

## Abstract

Objectives: Early-onset pancreatic cancer (EOPC) – defined as pancreatic cancer diagnosed before the age of 50 years – is associated with a poor prognosis as compared to later-onset pancreatic cancer (LOPC). Emerging evidence suggests that EOPC may exhibit a genetic signature and tumor biology that is distinct from that of LOPC. We review genetic mutations that are more prevalent in EOPC relative to LOPC and discuss the potential impact of these mutations on treatment and survival.

Materials and Methods: Using PubMed and Medline, the following terms were searched and relevant citations assessed: “early onset pancreatic cancer,” “late onset pancreatic cancer,” “pancreatic cancer,” “pancreatic cancer genes,” and “pancreatic cancer targeted therapy.”

Results: Mutations in *CDKN2*, *FOXC2*, and *SMAD4* are significantly more common in EOPC as compared to LOPC. In addition, limited data suggest that *PI3KCA* mutations are more frequently observed in EOPC as compared to LOPC. KRAS mutations are relatively rare in EOPC.

Conclusions: Genetic mutations associated with EOPC are distinct from those of LOPC. The preponderance of the evidence suggest that poor outcomes in EOPC are related both to advanced stage of presentation and unique tumor biology. The molecular and genetic features of EOPC warrant further investigation in order to optimize management.

## INTRODUCTION

Pancreatic cancer accounts for only 3% of all cancers diagnosed in the United States but is responsible for approximately 7% of all cancer-related deaths. Indeed, although relatively rare, pancreatic cancer represents the third most common cause of cancer-related death nationwide (https://www.cdc.gov/cancer/dcpc/research/update-on-cancer-deaths/index.htm) [1]. Incidence is increasing; it is projected that pancreatic cancer will become the second leading cause of cancer-related death by 2030 [2–4]. The reported frequency of early onset pancreatic cancer (EOPC) – defined here as pancreatic cancer diagnosed in patients under the age of 50 years – varies from 4% to 18% [5, 6], the majority of which are pancreatic ductal adenocarcinoma [7]. Although EOPC is less common than later-onset pancreatic cancer (LOPC), it contributes to a disproportionately high societal burden of potential years of life lost (PYLL), which has been estimated to be 25% in the United States and 40% in Europe [8]. Survival in patients with EOPC has improved over time but remains dismal [9].

There are no established data that definitively distinguish the tumor biology of EOPC from (LOPC). Previous studies have identified many genetic mutations that are shared between EOPC and LOPC [10–12]. However, genetic features that are unique to EOPC have also been described, including a low rate of *KRAS* and high rate of *SMAD4* mutations [13]. Based on the limited available data, the biology and behavior of EOPC may be significantly different from that of LOPC.

Similar to early-onset lung and breast cancer, EOPC is associated with inferior survival relative to LOPC [5]. Although late presentation may contribute to poor survival in EOPC, the role of genetic factors remains incompletely understood.

The purpose of this review is to identify genetic mutations that are more prevalent in EOPC as compared to LOPC and discuss the potential impact of these mutations on tumor biology, treatment, and survival.

### Risk factors, clinical presentation, and diagnosis of EOPC

The median age of diagnosis of pancreatic cancer in the United States is 71 years [14]. Risk factors for EOPC include smoking, obesity, diabetes mellitus, chronic pancreatitis, and a family history of pancreatic cancer [8, 15–17]. The effect of alcohol on pancreatic cancer risk varies based on age. However, it has been shown that excess alcohol use in individuals ≤45 years is associated with a markedly increased risk of both EOPC and LOPC [15].

**Figure 1:**
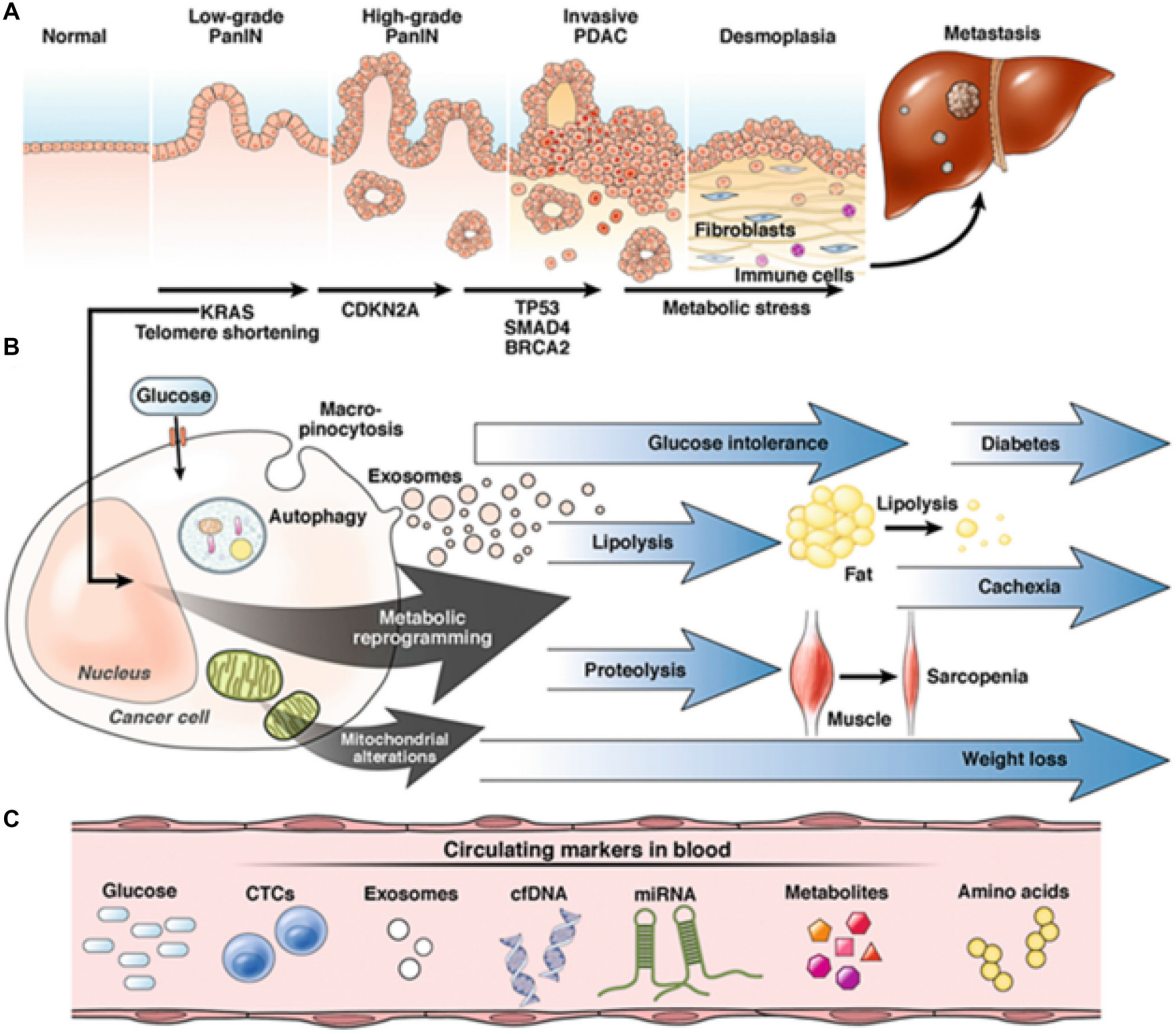
Progression of pancreatic adenocarcinoma (PDAC). (**A**) The progression of a normal pancreatic cell to PDAC begins with low-grade pancreatic intraepithelial neoplasia (PanIN); further mutagenesis – mediated by *KRAS*, *CDKN2A*, *TP53*, *SMAD4*, and *BRCA2* – leads to high-grade PanIN and subsequently invasive PDAC. PDAC is characterized by an exuberant desmoplastic reaction and signaling between fibroblasts and immune cells, which promotes epithelial-mesenchymal transition and ultimately metastasis. (**B**) *KRAS*-mediated metabolic reprogramming and mitochondrial alterations leads to a cascade of downstream events, manifesting with the triad of diabetes, cachexia, and sarcopenia classically associated with PDAC. (**C**) Cell-free DNA (cfDNA) may be used to identify metabolic aberrations during the initial stages of carcinogenesis and facilitate early diagnosis (Abbreviations: CTC: circulating tumor cells; miRNA: microRNA). Reprinted with permission from Søreide et al. [110].

The clinical presentation of EOPC and LOPC is identical and characterized by weight loss, jaundice, pruritus, nausea, and vomiting (Figure 1). EOPC has a predilection for the head of the pancreas while LOPC predominantly affects the tail [12]. Individuals with EOPC are more likely to present with advanced disease and are less likely to have multiple comorbidities as compared to their older counterparts [5, 6] (Table 1).

**Table 1:** Comparison of EOPC and LOPC

Variable	EOPC	LOPC
Clinical presentation	More advanced at time of diagnosis, present with more aggressive disease	Likely to present with early-staged disease
Median age of diagnosis (years)	46	71
*Gender difference*		
Male	More likely to be male	More common (1.3:1)
Female	Less likely to be female	Less likely to be female
Risk factors	**Tobacco, alcohol**, chronic pancreatitis, **Hereditary pancreatitis**, diabetes (type 1 and 2), BMI >40kg/m2, history of radiation therapy, cholecystectomy, gastrectomy	Tobacco, alcohol, chronic pancreatitis, diabetes (type 1 and 2), BMI >40kg/m2, history of radiation therapy, cholecystectomy, gastrectomy
Common genomic abnormality	**BRCA1/2**, SMAD4, KRAS, FOXC2, PI3KCA, CFTR, CDKN2a, c-MYC, **MICROSATELLITE INSTABILITY (MSI)**	BRCA1/2, SMAD4, **KRAS**, FOXC2, PI3KCA, CFTR, CDKN2a, c-MYC, MICROSATELLITE INSTABILITY (MSI)
Response to chemotherapy	May tolerate better and receive more aggressive therapy	Usually, poor candidate
Survival outcomes	Improves with more aggressive therapy, Median overall survival 8.6 months	Poor prognosis and less tolerant of surgery and systemic therapy, Median overall survival 8.0 months

Boldface = more common.

Next generation sequencing (NGS) is a “massive-parallel” or “high-throughput” sequencing technique used to determine the precise order of nucleotides within a strand of DNA/RNA. The first generation sequencing was developed by Frederick Sangers [18], who used two-dimensional chromatography to radiolabel base paired nucleotides. NGS represents a quantum leap in genetic analysis, facilitating millions of sequencing reactions run in parallel to allow for multiplexing of patient samples and thus simultaneous sequencing and detection of genetic mutations [19, 20]. NGS is now used in the analysis of both familial and sporadic pancreatic cancers [21].

### Genomic basis of EOPC

Although numerous studies have examined modifiable risk factors associated with EOPC [22], only recently with the widespread adoption of genome sequencing have the lesser-known genetic factors been investigated. Much of the recent research has focused on inheritable causes of EOPC, which have proven to be significant given that 10% of patients with these tumors have some genetic risk factor [23, 24]. Numerous systemic conditions and genetic mutations have also been shown to increase the risk of pancreatic cancer, including Peutz-Jeghers syndrome (*LKB1/STK11*), Li-Fraumeni syndrome (*TP53*), familial atypical mole-multiple melanoma (FAMMM) syndrome (*CDKN2A*), Fanconi anemia, and mutations in *BRCA1/2*, *CFTR*, *KRAS*, *SMAD4*, and *FOXC2* [6, 8, 25–27]. Notably, many of these genetic mutations are also associated with an increased risk of pancreatitis compared to the general population [28]. Several genetic mutations, including *BRCA1/2*, may be influenced by modifiable risk factors such as smoking and should be thought of in the context of the multistage theory of carcinogenesis.

There are only a few genetic mutations that have been linked specifically to EOPC in population studies, including *CDKN2A*, *FOXC2*, *SMAD4*, and *PI3KCA* [29–30]. Limited existing data are controversial but suggest that many of the genetic mutations associated with pancreatic cancer are shared between EOPC and LOPC. In a recent genomic and transcriptomic study by Raffenne et al., investigators compared mutational features of key genes and global methylation profiles between EOPC (defined as pancreatic cancer diagnosed at 55 years of age or younger) and late-onset pancreatic cancer (defined as pancreatic cancer diagnosed at age 70 or older). Authors concluded that the tumors in each group shared similar genetic and molecular features [31]. However, in another study by Bannon et al., investigators found a higher prevalence of germline mutations in EOPC. Individuals with EOPC also had better outcomes than those with LOPC, which was hypothesized to be related to superior DNA repair mechanisms and increased sensitivity to chemotherapy among younger patients [32].

**Figure 2:**
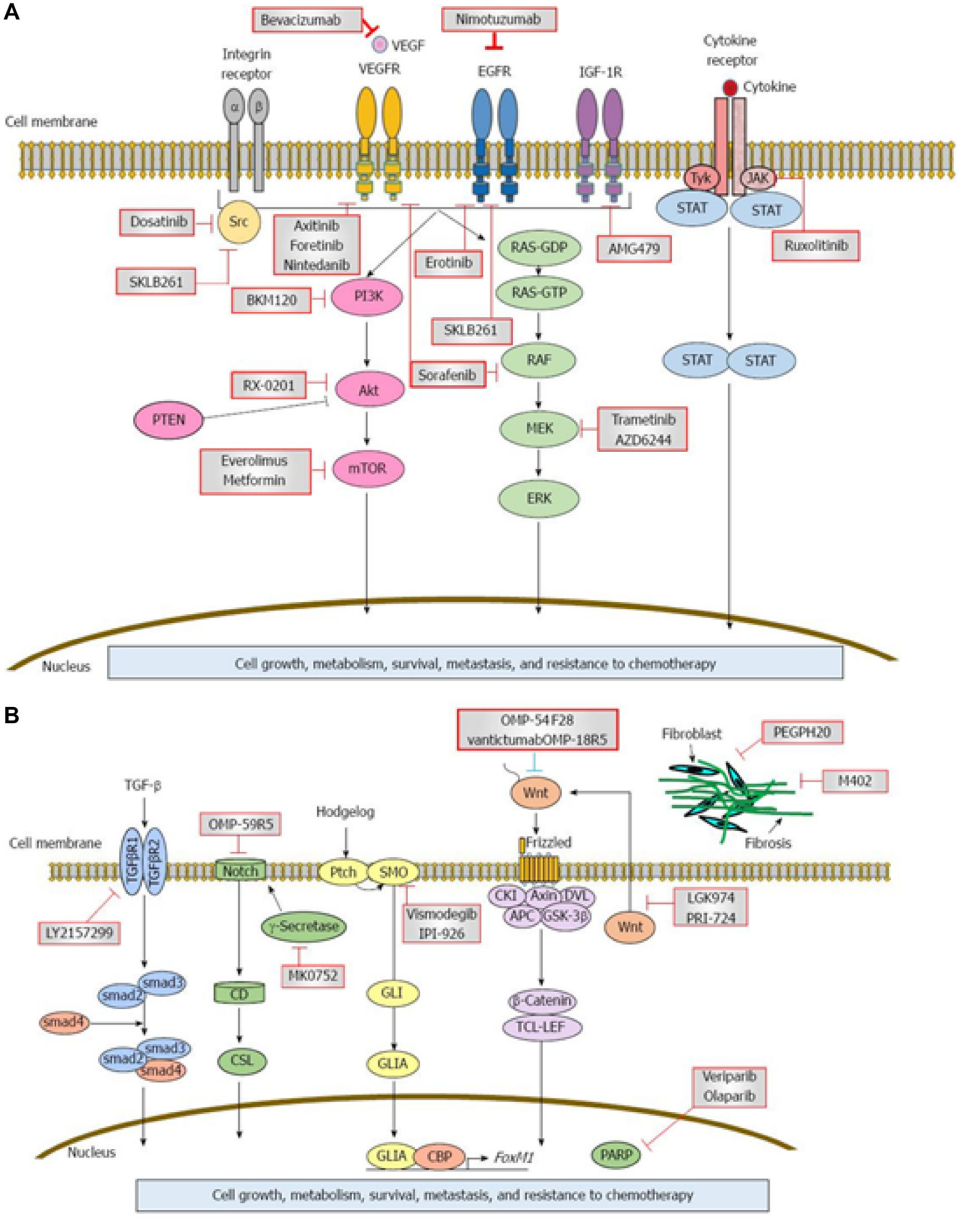
Signaling cascades and therapeutic inhibitors in pancreatic adenocarcinoma (PDAC). (**A** and **B**) Aberrant expression of multiple growth factors and growth factor receptors as well as intracellular, extracellular, and intranuclear proteins is implicated in the pathogenesis of PDAC. Examples include vascular endothelial growth factor (VEGF), epidermal growth factor receptor (EGFR), insulin-like growth factor-1 receptor (IGF-1R), gamma-secretase, and poly (ADP-ribose polymerase (PARP). Each abnormal protein may serve as a target for monoclonal antibodies or chemotherapeutic agents, which inhibit abnormal protein activity and can potentially reduce the rate of disease progression. Circles indicate critical signaling pathways and red squares indicate potential targets. Reprinted with permission from Matsuoka et al. [109].

The mechanism of pancreatic carcinogenesis involves a complex interplay of multiple related genetic and molecular factors (Figure 2). Other genetic mutations are discussed below.

### Kirsten rat sarcoma virus (*KRAS*) mutation

*KRAS* mutations are present in approximately 90% of pancreatic ductal adenocarcinoma [33, 34]. However, *KRAS* mutations are relatively rare in EOPC. In one recent study, authors found that only 42.8% of patients with EOPC expressed a *KRAS* mutation [10]. The common types of KRAS are on exon 2 codons 12 and 13 with relative frequency of 71–80% [35, 36] and mostly located at G12C, G12D and G12R in pancreatic cancer [37, 38]. There is no data on frequency in EOPC verses LOPC.

**Figure 3:**
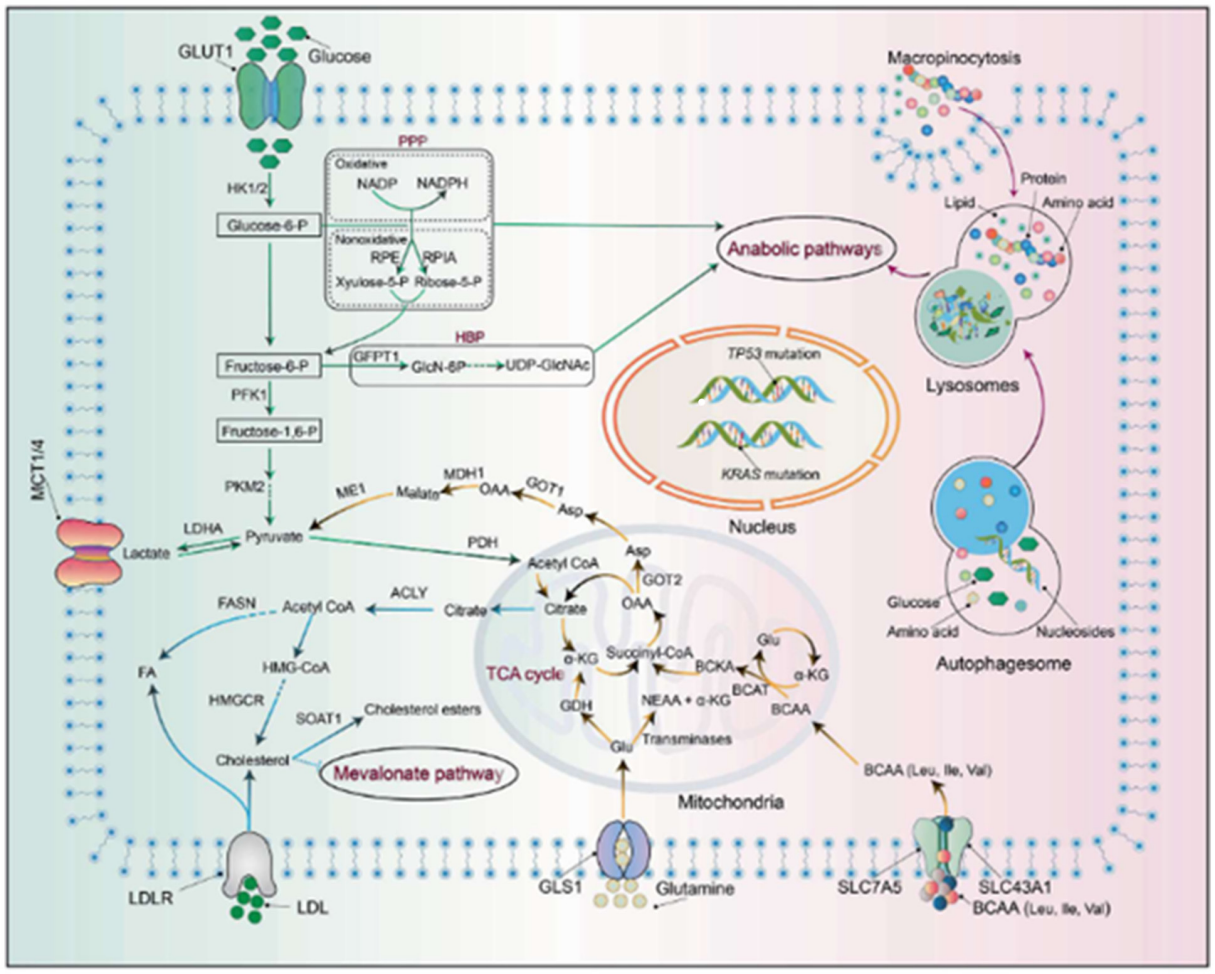
Metabolic reprogramming in pancreatic adenocarcinoma (PDAC). Activation of *KRAS* and mutations of *TP53* results in a cascade of downstream effects that generates biosynthetic precursors to drive various anabolic pathways, including the non-oxidative arm of the pentose phosphate pathway (PPP) and the hexosamine biosynthesis pathway (HBP). In addition, *KRAS* activation affects glutamine metabolism, increasing the NADPH/NADP^+^ ratio and increasing turnover of glutathione (GSH) via reduction of its oxidized form. Branched chain amino acid (BCAA) catabolism is also catalyzed in the setting of *KRAS* activation via increased BCAT2 (branched chain amino acid transaminase 2) activity. The ultimate outcome of these aberrant pathways is enhanced nutrient salvaging and further promotion of neoplastic processes. Reprinted with permission from Wang et al. [110].

Interestingly, mathematical models have shown that it takes nearly twelve years from the oncogenic event that initiates pancreatic carcinogenesis until the development of the parental clone and an additional seven years for the development of metastatic subclones within the primary cancer [39, 40]. Therefore, most *KRAS* mutant pancreatic cancers could be considered EOPC if detected via early screening. Screening for early detection in high-risk individuals such as familial pancreatic cancer could potentially identify more patients with pancreatic cancer at an early stage and age [41]. The mechanism of *KRAS* mutation-associated carcinogenesis is illustrated in Figure 3.

### Microsatellite instability (*MSI*)

Mutations or epigenetic changes of mismatch repair genes such as *MLH1, MSH2, MSH3, MSH6, PMS1, and PMS2*, which repair DNA replication errors, result in MSI [42]. The presence of MSI is associated with a favorable prognosis in colorectal cancer [43], gastric cancer [44], and cancer of papilla of vater [45]. However, MSI-positivity is associated with a poor prognosis in breast cancer [46, 47] and non-small cell lung cancer [48]. The prevalence of MSI- positivity ranges from 3–67% in pancreatic cancer [49–53]; similar to other gastrointestinal malignancies, MSI-positivity is associated with a favorable prognosis [49, 54]. There is a paucity of data on the prevalence of MSI in EOPC. Bergmann et al. assessed molecular characteristics of 7 patients under 40 years and found mismatch repair gene products MLH1, MSH2 and MSH6 to be present in all patients [10]. There are no data specific to the prevalence of MSI positivity and survival among patients with EOPC.

### Cyclin dependent kinase inhibitor 2A(*CDKN2A*)

One of the recently studied genes associated with EOPC is *CKDN2A*, which normally functions as a tumor suppressor gene involved in the pathway that inhibits the cell cycle at the G1 checkpoint. Mutations in *CDKN2A* are found in 95% of pancreatic cancers [55, 56]. *CKDN2A* encodes the protein p16inka, which acts as a cell cycle regulator. Previous studies have shown that loss of the p16inka protein may increase progression of cells that have a pre-existing *KRAS* mutation [57]. Notably, however, *KRAS* mutations prevalence appear to be less common in EOPC as compared to LOPC [29, 30]. Tsang and colleagues found that a biallelic *CDKN2A* mutation was significantly increased in EOPC patients [30]. Targeting different proteins in this pathway has been investigated in a few prior studies with limited clinical efficacy to date.

### Forkhead box protein C2 (*FOXC2*)

*FOXC2* is an oncogenic transcription factor associated with many different cancers, including hepatocellular carcinoma and breast cancer. The true prevalence of *FOXC2* mutations in pancreatic cancer remains to be established. It is upregulated in pancreatic ductal adenocarcinoma and enhances growth and migration of cancer cells [58]. It interacts with beta-catenin and promote cell growth by activating beta-catenin/T-cell factor 4 signaling. The beta-catenin/T-cell factor 4 interaction may serve as a therapeutic target for future drug development [58, 59]. Tsang and colleagues showed that *FOXC2* upregulation was associated with EOPC and potentially early epithelial-to-mesenchymal transition [30].

### SMAD4

*SMAD4* is an important tumor suppressor gene that is found to be inactivated in approximately 50% of pancreatic cancers and has been associated with a more aggressive clinical course [5, 10, 60, 61]. In addition to its tumor suppressor function, it also acts in part of the transforming growth factor beta (TGF-β) pathway. In the setting of EOPC, *SMAD4* serves as part of this pathway, where it forms as complex with specific sequences of DNA in the cell nucleus after it is activated by a TGF-β protein. When *SMAD4* is mutated, the cell proliferation process is left unchecked and rapid cell growth follows. Previous analyses have demonstrated that patients with EOPC had higher mutations rates of *SMAD4* than those with LOPC [29].

### PI3KCA

*PI3KCA* is a gene that encodes the p110 alpha protein which is involved in the phosphatidylinositol 3-kinase (PI3K) pathway. Mutations are present in 3–5% of patients with pancreatic cancers [62–64]. This frequently mutated oncogene has been previously studied and implicated in a number of cancers [65]. However, it is only recently that it has been linked to EOPC. Ben-Aharon et al. found that this gene was more often implicated in EOPC than in LOPC [29]. Furthermore, this pathway has been studied with regards to the *PTEN* tumor suppressor gene that normally inhibits this pathway. Previous studies have shown mutations to *PTEN* result in accelerated mutated pancreatic cell growth but have so far not been linked to EOPC [66].

### CFTR

Cystic fibrosis transmembrane conductance regulator (*CFTR*) is a transmembrane chloride channel that helps maintain fluid homeostasis in the body. Mutations in the form of phenylalanine 508 deletion (ΔF508) are the most commonly seen alteration. The prevalence of CFTR mutations in pancreatic cancer is estimated range between 5 and 8% [67, 68]. A potential link between *CFTR* mutations and EOPC was proposed over a decade ago. In 2005, McWilliams and colleagues found that 8.4% of EOPC patients had *CFTR* mutations as compared to 4.1% in the control group [68]. However, this study was limited by its very small size (*n* = 33) with only two patients found to have a *CFTR* mutation; these patients were 42 and 50 years of age. A follow up study by the McWilliams group again found a significant increase in pancreatic cancer in carriers of the *CFTR* gene (5.3% versus 3.8%) along with a younger average onset (62 versus 67 years) [67]. Other studies have described variable findings on the influence of *CFTR* mutations in early pancreatic cancer, including a recent analysis in which investigators found no significant difference in the rate of EPOC among patients with the most common *CFTR* variants (ΔF508 mutation and 5T allele) as compared to a control group [69].

### BRCA1/2

*BRCA1/2* are tumor suppressor genes that are well known for their role in breast and ovarian cancers. They have also been described in pancreatic cancers. Pathogenic *BRCA2* and *BRCA1* mutations are found in approximately 2%, and ≤1% of pancreatic cancers, respectively [70–72]. In a 450 patient cohort of patients ≤50 years of age, 114 of whom underwent germline testing, 29% had pathologic germline alterations and nearly 16% of those patients demonstrated *BRCA1/2* mutations [73]. However, other studies have found no significant increase in *BRCA1/2* mutations among EOPC cohorts when compared to those with LOPC [29].

### Treatment and survival of EOPC

#### Surgical treatment

Surgical treatment of pancreatic cancer is the only potentially curative modality when diagnosed at early stage of disease. Unfortunately, however, pancreatic cancer is most often diagnosed at an advanced stage [5]. Contraindications to surgery include metastasis to liver, omentum, peritoneum, or any other extra-abdominal sites, occlusion of the superior mesenteric artery or encasement of more than half of vessel circumference, and involvement of the inferior vena cava [74]. Stage II pancreatic cancer – defined as local tumor that has grown outside the margins of the pancreas but without vascular invasion – may be amenable to resection [75]. Individuals presenting with later stage cancers are typically treated with chemotherapy.

#### Medical treatment

Limited data suggest that EOPC is more aggressive and is associated with poorer survival than LOPC [5, 6, 76]. However, as of this writing, there are no large-scale studies comparing the outcomes between EOPC and LOPC; available studies are small and predominantly retrospective.

The prognosis of pancreatic cancer is dismal, with a 5-year survival rate of 8%, but modest gains have been made over the last decade with a gradual increase in median survival [77]. Treatment of early-staged pancreatic cancer is surgical resection [78, 79]. Improvements in survival have largely been attributable to novel targeted therapies in advanced and metastatic setting (Figure 2). Furthermore, increasing the use of germline testing, utilizing molecular tumor analysis, and employing adjuvant therapy with modified FOLFIRINOX (leucovorin, 5- fluorouracil, irinotecan, oxaliplatin) have all contributed to prolonged survival for individuals with pancreatic cancer [80].

As compared to patients with LOPC, individuals with EOPC are more likely to present with advanced stage disease. Notably, although patients with EOPC are more likely to undergo chemo- and radiation therapy and surgery as compared to their counterparts with LOPC, several studies suggest they nevertheless face poorer outcomes [5, 6]. Other studies have reported opposite or neutral findings regarding survival [76, 81–83].

Unfortunately, there are no randomized controlled trials (RCTs) examining management strategies and survival differences between EOPC and LOPC; data from existing studies can only be extrapolated to provide insight into the potential effect of available therapeutics on EOPC. For example, gemcitabine was compared to FOLFIRINOX in one RCT involving 342 patients aged 65 years or younger with metastatic pancreatic cancer and good performance status [84]. The FOLFIRINOX group was noted to have a significant survival advantage, albeit with increased toxicity, over the gemcitabine group. The hazard of death was 0.61 (95% CI; 0.46–0.82) for those ≤65 years and 0.48 (95% CI; 0.30–0.77) for individuals older than 65 years. In another RCT involving 493 patients [85] with pancreatic cancer status-post resection, and had no evidence of metastatic disease, malignant ascites, or pleural effusion were eligible for inclusion. In this study, 59.2% were ≤65 years. Patients who received a modified FOLFIRINOX regimen showed superior disease free and overall survival as compared to those treated with gemcitabine. In subgroup analysis, there was no significant difference in outcomes based on age. Given the relatively high rate of grade 3 and 4 adverse effects, FOLFIRINOX is currently recommended only for patients with good performance status. Patients with EOPC typically have fewer comorbidities than those with LOPC and are therefore more often deemed candidates for FOLFIRINOX therapy. Moreover, EOPC may demonstrate biology and behavior that is distinct from that of LOPC. Consequently, the response to available therapies may differ between the two clinical entities [60].

Approximately 10% of pancreatic cancers have familial inheritance [83, 86]. Screening first-degree relatives of individuals with multiple family members affected by pancreatic cancer can help identify disease precursors, as there are numerous syndromes associated with pancreatic cancer, including Peutz–Jeghers syndrome, p16, BRCA and hereditary non-polyposis colorectal cancer (HNPCC) [41, 57, 87]. The recent POLO trial investigated the effect of maintenance Olaparib therapy on individuals with germline *BRCA*-mutated metastatic pancreatic cancer that had not progressed during platinum-based chemotherapy. Subjects included in the study were relatively young, with median age of 57 years. Authors concluded that maintenance therapy with Olaparaib increased progression-free survival as compared to placebo [88]. Moreover, there was no statistically significant difference in the incidence of grade 3 or higher adverse events (40% in the Olaparib group versus 23% in the placebo group). In a prespecified subgroup analysis of progression-free survival, investigators demonstrated that outcomes were most improved among the youngest patients included in the cohort. The effect of poly ADP ribose polymerase (PARP) inhibitors in *BRCA*-mutated EOPC as a first-line treatment for metastatic disease warrants further exploration.

The response to treatment may vary in patients with both a germline *BRCA1/2* and *PALB2* (gBRCA/PALB2+) mutation. In a phase II trial comparing cisplatin and gemcitabine with or without veliparib in gBRCA/PALB2+ in patients with stage III or stage IV pancreatic adenocarcinoma, cisplatin and gemcitabine were shown to be the more effective regimen and concurrent veliparib did not improve response rate [89]. However, the median age of subjects was 64 years; it is conceivable that individuals with EOPC which are likely to have some genetic mutation [90–92] may likely benefit from the addition of veliparib. Similarly, in patients with metastatic pancreatic adenocarcinoma, nab-paclitaxel plus gemcitabine significantly improved overall survival, progression-free survival, and response rate compared with gemcitabine alone [93]. In subgroup analysis of same study, overall survival in patients <65 years old (majority; 57.6%) overall survival was superior (Hazard ratio; 0.65, 95% CI:0.53–0.79) versus older patients (Hazard ratio; 0.81, 95% CI:0.63–1.03).

Molecular targets, such as human epidermal growth factor receptor type 1 (HER1/EGFR), which are overexpressed in many pancreatic cancers have also been targeted clinically with mixed outcomes. Moore et al. first demonstrated a statistically significantly improvement in survival in advanced pancreatic cancer by adding erlotinib to gemcitabine [94]. In the Moore group study, the hazard ratio for survival was significant for patients ≤65 years (HR = 0.75 (0.58 to 0.96), but not for those > 65 years old. However, no difference was noted on stratifying by EGFR status. However, Hammel et al. conducted a phase 3 trial involving patients with locally advanced pancreatic cancer with disease controlled and did not find difference in overall survival with gemcitabine when compared with gemcitabine plus erlotinib as maintenance therapy [95]. The median age in the Hammel group study was older at 63 years (interquartile range of 58–71 years). Similarly, in a phase II trial by Abrams et al. showed addition of adjuvant erlotinib to gemcitabine did not show any clinical benefit [96]. Clinical trials to specifically look at effects of specific actionable molecular targets in EOPC is warranted.

Circulating tumor DNA (ctDNA) can be used to determine pancreatic adenocarcinoma prognosis and can conceivably help select targeted therapeutic agents [97]. Indeed, in a 2018 study by Riviere et al., investigators used ctDNA to identify patients with genomic alterations that were potentially actionable by experimental or approved drugs [98]. Furthermore, ctDNA can be used to identify germline mutations and may serve as an adjunct to traditional hereditary cancer gene testing [99]. The use of ctDNA in pancreatic adenocarcinoma diagnosis, prognosis, and treatment is rapidly expanding. Future large-scale analyses of ctDNA may be used to identify distinct genomic alterations and therapeutic targets among patients with EOPC.

Immune checkpoint inhibitors (ICIs) have historically been ineffective for the management of pancreatic adenocarcinoma. However, emerging evidence suggests that ICIs may play an important role in the management of both EOPC and LOPC. In a 2021 study by Lenzo et al., authors described several subsets of patients who may benefit from ICI therapy, including those with a PD-L1-driven phenotype or PD-1 axis-driven immunosuppression [100]. A subsequent study by Botta et al. showed that a small subset of patients with genomic alterations in SWItch/Sucrose Nonfermentable (SWI/SNF) chromatin remodeling genes may be responsive to ICIs; authors postulated that patients with SWI/SNF mutations demonstrate increased sensitivity to T cell-mediated cytotoxicity [101]. Future prospective studies may be of value to determine which patient populations are most likely to benefit from ICI therapy.

### Summary of treatment recommendations for EOPC

There are no established treatment guidelines tailored toward EOPC. In advanced or metastatic disease, treatment for EOPC and LOPC is similar and based predominantly on performance status and comorbidities [102]. The first-line systemic treatment options are FOLFIRINOX or gemcitabine/nab-paclitaxel [84, 93]. A direct comparison between the two regimens reveals that FOLFIRINOX offers prolonged overall and progression-free survival as compared to gemcitabine/nab-paclitaxel. However, while there are no data on quality of life (QoL) for gemcitabine/nab-paclitaxel, data showed FOLFIRINOX showed a delay of deterioration of health status [103]. There are no clinical trials which directly compare FOLFIRINOX(FFN), nab-paclitaxel plus gemcitabine in unresectable tumor setting. In a study using real word experience, 225 patients received either FOLFIRINOX, gemcitabine plus nab-paclitaxel, or gemcitabine alone. Those who received FFN were more likely to be younger, and additionally, those receiving FFN or gemcitabine plus nab-paclitaxel had better overall survival than gemcitabine alone [104]. The second line options include gemcitabine, gemcitabine plus cisplatin, and gemcitabine/nab-paclitaxel [105].

Challenges in medical management of pancreatic cancer are vast and are related to metabolism of therapeutic agents [106], management of the inflammatory response [107], and the complex immunological milieu which leads to fibrotic reaction that promotes blockade of active immune cells [108]. Given the poor prognosis of pancreatic cancer, it is more pressing to examine unique features of EOPC that may lead to the identification of new molecular targets and the development of new therapeutics.

## CONCLUSIONS

Pancreatic cancer is increasing in incidence and will become second leading cause of cancer death by 2030. Unfortunately, the prognosis of pancreatic cancer remains dismal. Individuals with EOPC tend to have fewer comorbidities and are more likely to undergo chemo- and radiation therapy as compared to their counterparts with LOPC, but nevertheless face a worse prognosis. Poor outcomes in EOPC patients may be multifactorial and related to advanced stage of presentation at diagnosis and unique tumor biology. The genomic basis of EOPC warrants further investigation and consideration as a distinct clinical entity. Future studies and trials may lead to the identification of genetic mutations distinct to EOPC and the development of new targeted interventions.

## Abbreviations

EOPC: early-onset pancreatic cancer
LOPC: later-onset pancreatic cancer
MSI: microsatellite instability
